# User perceptions of surgical antimicrobial prophylaxis guidelines in orthopaedic surgery in a tertiary Australian hospital

**DOI:** 10.1371/journal.pone.0319829

**Published:** 2025-03-20

**Authors:** Sarah Hassan, Vincent Chan, Julie E. Stevens, Ieva Stupans, Juliette Gentle

**Affiliations:** 1 Pharmacy, School of Health and Biomedical Sciences, RMIT University, Bundoora, Victoria, Australia; 2 Department of Orthopaedics, Northern Health, Epping, Victoria, Australia; 3 Clinical and Health Sciences, University of South Australia, Adelaide, South Australia, Australia; 4 Adelaide Medical School, Faculty of Health and Medical Sciences, University of Adelaide, Adelaide, South Australia, Australia; 5 Department of Surgery, University of Melbourne, Epping, Victoria, Australia; University of KwaZulu-Natal College of Health Sciences, SOUTH AFRICA

## Abstract

**Background:**

Surgical antimicrobial prophylaxis remains the most common indication for antimicrobial use in Australian hospitals. Despite efforts to improve practice, adherence to guideline recommendations continues to be suboptimal across surgical disciplines, including orthopaedics. The *Therapeutic Guidelines: Antibiotic* v16 currently advocates for single dose prophylaxis for open reduction internal fixation (ORIF) procedures. Audits undertaken in one Australian tertiary hospital have identified low levels of adherence to this recommendation. It is unclear as to why guidelines are not adhered to in this setting.

**Aim:**

To understand the factors that influence multidose prescribing for ORIF procedures and the barriers and enablers to guideline use in an Australian tertiary hospital.

**Materials and methods:**

Interviews (focus groups and one-on-one sessions) were held with orthopaedic surgeons (consultants), orthopaedic registrars, pharmacists, and anaesthetists from a tertiary public hospital in Australia. The Theoretical Domains Framework (TDF) was used to analyse results.

**Results:**

Six focus groups and three one-on-one interviews were conducted. Data were mapped to 12 TDF domains. Although clinicians were aware of guideline recommendations, this alone did not encourage the use of single dose prophylaxis. The decision to prescribe postoperative antibiotics was influenced by a combination of patient and environmental factors as well as fear of infection development. The lack of guideline specificity was commonly highlighted as a barrier to guideline use, as well as lack of agreement with guideline content. Enablers to guideline use included education that was targeted and repetitive, as well as improved dissemination of guidelines.

**Conclusion:**

There are myriad factors that influence the decision to prescribe postoperative antibiotics for ORIF procedures. By understanding the social and cultural context of a local setting and the barriers and enablers that pertain to an environment, interventions can be developed to enhance guideline use, thereby improving antimicrobial prescribing.

## Introduction

In Australia, elective orthopaedic surgeries are some of the most commonly performed procedures, accounting for 13.4% of all elective procedures performed in 2021-22 [1]. National audits have previously identified that prescribing for surgical antimicrobial prophylaxis (SAP) in orthopaedic surgery is inappropriate in the procedural (i.e., preoperative and intraoperative) and post-procedural (postoperative) setting [2,3]. Preoperatively, this can be attributed to SAP being administered at the incorrect time whilst postoperatively, this can be due to SAP being administered when not required or administered for the incorrect duration for procedures in which SAP is recommended [2].

National SAP prescribing recommendations can be found in the *Therapeutic Guidelines*, an Australian resource that covers disorders commonly seen in clinical practice [4]. The *Therapeutic Guidelines* are written by independent expert groups comprising of local clinicians who are specialists in their fields, general practitioners, pharmacists, junior hospital doctors and in-house editors [4]. Locally endorsed (institutional) guidelines are often designed based on *Therapeutic Guidelines* recommendations and can be modified where necessary according to local antimicrobial resistance patterns or certain patient demographics [5].

Current guidance for SAP in orthopaedic surgery indicates that a single preoperative antibiotic dose is sufficient to prevent infections postoperatively [6–8], with single dose prophylaxis unlikely to be inferior to multidose prophylaxis in preventing surgical site infections [9]. Despite the available evidence regarding how antibiotics can be prescribed in the orthopaedic setting, adherence to such recommendations has been identified as inadequate [2,3,10–13].

Open reduction internal fixation (ORIF) procedures are a type of orthopaedic surgery that is often performed; however, it has been identified that SAP prescribing in this setting is not in line with guideline recommendations [2,3,10,13]. Although interviews have been performed with stakeholders from a range of surgical disciplines to understand the complexities of SAP decision making [14–19], to the best of our knowledge, little has been conducted focusing solely on the orthopaedic setting.

Reasons for guideline non-adherence in the surgical setting have been identified and include a combination of personal and environmental or organisational factors [20–23]. Personal factors can relate to guideline knowledge, hierarchical relationships influencing decision making and fear of complications, while organisational factors can relate to workflow, inaccessibility of local guidelines and social norms dictating practice [20–23]. The use of a framework can help to better understand these factors, with one commonly used framework in the healthcare setting known as the Theoretical Domains Framework (TDF) [24]. The TDF can be used to understand and evaluate implementation problems as well as aid in intervention design [24] and has previously been used to understand the barriers and enablers to SAP guideline adherence [20], as well as to design interview guides to understand the factors that determine antibiotic prescribing [25].

There is a need to understand the various factors that influence prescribing in the orthopaedic setting as it provides an opportunity to change practice and limit excessive antibiotic use in a high-volume area. To understand this phenomenon further, we held interviews with key users of SAP guidelines to understand the factors that determine prescribing practice and guideline adherence, using ORIF procedures as an example to illustrate this in a tertiary Australian hospital where low levels of guideline adherence had previously been identified [10,13]. The recent introduction of local SAP guidelines at this hospital also provides an opportunity to further investigate stakeholder views on the impact of local guideline introduction and dissemination on practice.

## Materials and methods

### Participants and recruitment

Orthopaedic surgeons (consultants), orthopaedic registrars, surgical pharmacists (with prior experience on an orthopaedic ward) and anaesthetists were invited to participate in this study at a tertiary hospital in Melbourne, Australia. Purposive sampling was used to recruit participants. Contact was initially made with the head of department for each specialty (orthopaedics, pharmacy, and anaesthesia), with recruitment flyers and participant information sheets disseminated via email to introduce the project. This information was then forwarded to members of the department by the administrators or research coordinators for each specialty.

Reminder emails were sent to each department at least 4 weeks after the time of initial contact, with all recruitment taking place between March and August 2022. An effort was made to include orthopaedic ward nurses in the project, however due to staff shortages and difficulty recruiting during the COVID-19 pandemic, a decision was made to exclude nursing staff from the project.

### Ethics statement

Ethics approval to conduct this study was granted by Northern Health’s Office of Research, Ethics and Governance (NLR 72459), and registered with RMIT University’s College Human Ethics Advisory Network (RM 24642). All participants provided written, informed consent prior to participating in the study.

### Data collection

An interview guide (S1 Table) was developed by the primary author (SH – pharmacist and PhD candidate at time of development), which was reviewed by co-authors (VC, JS, IS – academics and PhD holders), all of whom have experience with conducting qualitative research. Questions were also discussed and piloted with a senior orthopaedic surgeon (JG) prior to interviews to ensure questions were suitable and relevant to the clinical setting. The TDF [24] was used as the foundation for question design, with representative domains selected from ones previously identified in the literature [14,20]. Questions were modified to suit each participating discipline and aimed to gather an understanding of the role each specialty has when dealing with SAP, the factors that influence postoperative prescribing for ORIF procedures as well as the barriers and enablers to using SAP guidelines (both local and national). For context, local SAP guidelines were introduced at the hospital in 2021 and contains reference to a range of surgical procedures, including ORIFs. Recommendations pertinent to orthopaedic surgery were developed by the Antimicrobial Stewardship/Infectious Diseases team in collaboration with senior members of the Orthopaedics department, with guidelines then disseminated through various means (details of guideline design and dissemination can be found in the referenced article) [13].

Identifiable information (i.e., participant details) were accessible to SH, who was responsible for contacting participants and conducting interviews. No prior relationship existed between the participants and SH. Participants were briefed on the goals of the study prior to conducting the interviews, with pseudonyms allocated to all participants. Focus groups were chosen as the primary method of interview as a vast amount of detailed information can be collected in a short period of time [26]. This is particularly useful in a hospital setting where the time of clinical staff is limited by work demands, with restricted timing heightened due to the COVID-19 pandemic. In situations where participants could not attend a focus group session, one-on-one interviews were also held. Interviews were estimated to take between 20 and 30 minutes to complete and were conducted at mutually convenient times between SH and the participants, both during and outside of participant work hours. Each focus group session consisted of a single group of healthcare professionals and interviews with orthopaedic consultants and orthopaedic registrars were also performed separately to allow freedom of expression during the sessions. No incentives were provided to participants.

Video conferencing software (Microsoft Teams, Microsoft 365) was used to conduct, audio-record and transcribe all interviews, with interviews conducted virtually. Transcripts were subsequently reviewed and edited for accuracy by SH. Interviews continued until data saturation was reached. Repeat interviews were not conducted, and transcripts were not returned to participants for review as interviews were held during an extremely busy period at the hospital.

### Data analysis

Initial interviews were analysed to identify emerging concepts and to allow for refinement of later interviews. A thematic analysis approach was used to analyse data [27]. All interviews were read multiple times to gain a deeper understanding of the content before beginning the analysis. Codes were then independently developed by SH, VC, JS and IS for two interviews, with a codebook shared amongst authors. Codes were refined after group discussion, with subsequent coding completed by SH. Codes were derived both inductively and deductively, with emerging themes identified and sub-themes mapped to the relevant domains of the TDF. The TDF was used as the framework of choice for analysis and reporting of data as it can be used to understand the factors that influence healthcare professional behaviours in relation to implementing evidence-based recommendations [24]. NVivo (released in March 2020) [28] was used to organise data and to assist with analysis. Study findings are reported according to the Consolidated Criteria for Reporting Qualitative research (COREQ) checklist (S2 Table) [29].

## Results

**Fig 1:**
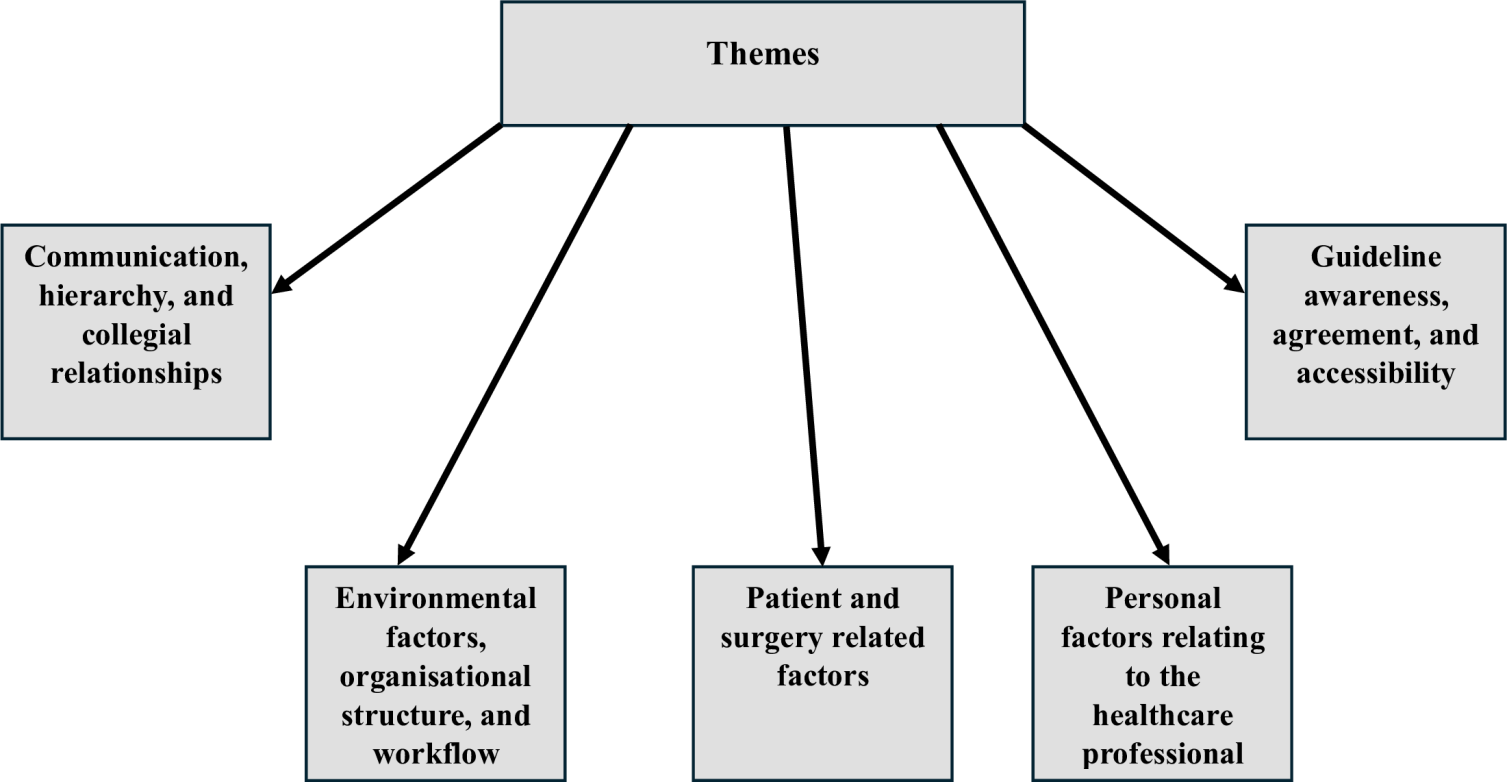
Major themes identified that highlight the factors that influence prescribing for ORIF procedures.

**Fig 2:**
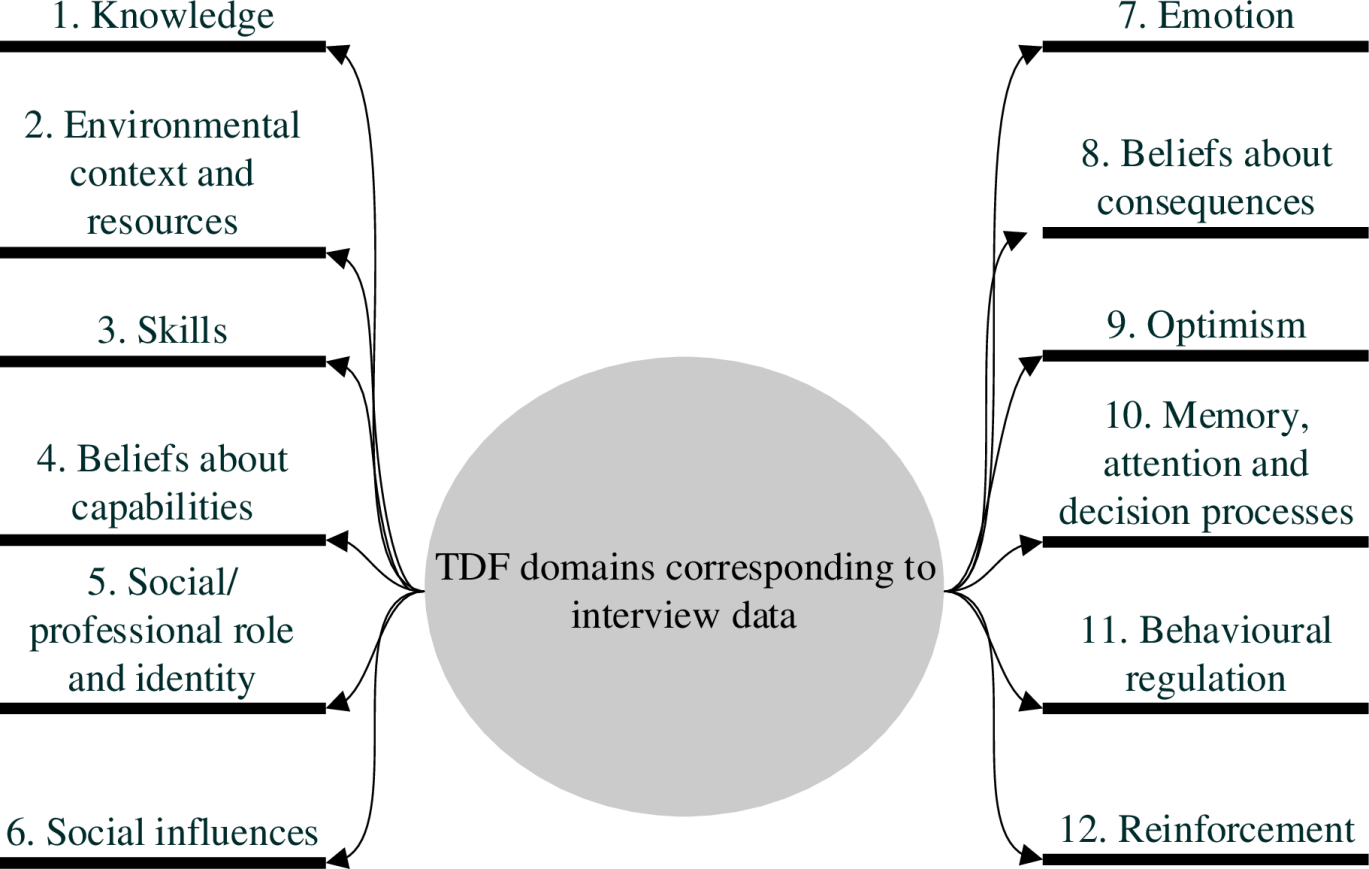
TDF domains representing the factors that influence SAP decision making and adherence to guidelines.

Six focus groups and three one-on-one interviews were conducted, with a total of 28 participants. Interview and participant characteristics are found in Table 1. Interviews ranged from 20 to 41 minutes in duration. Five major themes were identified (Fig 1). Sub-themes were then mapped to 12 TDF domains (Fig 2): knowledge; environmental context and resources; skills; beliefs about capabilities; social/professional role and identity; social influences; emotion; belief about consequences; optimism; memory, attention and decision processes; behavioural regulation; reinforcement. Illustrative quotes are used to demonstrate participant perceptions of SAP guidelines and the factors that influence prescribing for ORIF procedures.

**Table 1:** Interview and participant characteristics.

	Participants (n = 28)
**Participant characteristics**	
**Role**	
Anaesthetists	7
Orthopaedic Consultants	11
Orthopaedic Registrars	5
Pharmacists	5
**Length of practice (years)**	
0–5	15
6–10	6
11–15	5
16–20	1
More than 20	1
**Interview characteristics**	
** Focus groups**	
FG1 (Orthopaedic Registrars)	3^a^
FG2 (Orthopaedic Consultants)	3^a^
FG3 (Orthopaedic Consultants)	4
FG4 (Orthopaedic Consultants)	3^a^
FG5 (Pharmacists)	5
FG6 (Anaesthetists)	7
** One-on-one interviews**	
INT.1-2 (Orthopaedic Registrars)	2
INT.3 (Orthopaedic Consultants)	1

^a^Limited to three participants only due to participant availabilities.

### Domains 1 and 2: ‘Knowledge’ (1) and ‘Environmental context and resources’ (2)

#### 1.1. Awareness.

Knowledge and awareness of SAP guideline recommendations were variable across the board, with some participants aware of the recommendation for single dose prophylaxis for ORIF procedures, whilst others were unaware of this change (S3 Table). However, awareness of guidelines does not equate to adherence as clearly stated by one participant:

*“So, we’re aware of them, but we don’t necessarily use them.”* (Orthopaedic Consultant A)

Participants highlighted the importance of receiving education when a new guideline is introduced, particularly as not all healthcare professionals have the opportunity to review guideline updates due to competing demands.

*“I was aware that they came out... but we have not had any education on it.”* (Anaesthetist A)

*“If no one brings that to our attention, I can assure you we’re not all reading PROMPT [document management system where guidelines are located] as bedtime reading just to see what changes there have been made.”* (Anaesthetist F)

#### 1.2. Guideline agreement.

The extent to which guidelines are followed can also be impacted by whether or not a healthcare professional agrees with its contents or finds it suitable to their practice (S3 Table).

*“I would try to go along with them as much as possible as long as I felt that they were reasonable…If I had concerns about what the suggestion was, I wouldn’t follow the guideline.”* (Orthopaedic Consultant B)

*“There’ll be times where I disagree with the guidelines as well. And especially in revision surgery or worried about infection in arthroplasty, then I might do something different to what the guidelines suggest.”* (Orthopaedic Consultant D)

#### 2.1. Guideline specificity.

Furthermore, the lack of guideline specificity was commonly reported as a barrier to guideline adherence (a range of barriers and enablers to using SAP guidelines can be found in Table 2). Guidelines were often viewed as documents not written from a surgical angle, limiting its applicability to the day-to-day practices of a surgeon.

**Table 2:** Barriers and enablers to guideline use and associated TDF domains.

TDF Domains	Barriers	Illustrative quotes
Environmental context and resources	Lack of guideline specificity	“That [local] guideline doesn’t take orthopaedic specialty as the focus of antibiotic prophylaxis. It talks about surgical prophylaxis. The worry in orthopaedic is slightly different because of the implant insertion and the nature of a lot of operations that we do with prolonged operation time, soft tissue dissection etc.” *(Orthopaedic Consultant C)*
		“It needs to be more specific. ‘Cause [sic] I think they’re quite general…but then I guess by their nature, you can’t really make guidelines too specific, otherwise it wouldn’t really do their job. So, it’s difficult.” *(Orthopaedic Consultant G)*
		“The problem with the current guidelines is they’re not tailor made to every situation.” *(Orthopaedic Consultant H)*
		“The issue with things like antibiotic guidelines is they have to be broadly applicable, which means they’re not very specific to any individual case. And the issue will be you’ll want specifics for the specific case you’re looking at hence, why you’re looking up the guideline.” *(Orthopaedic Registrar E)*
	Flow chart medicine	“Guidelines to me, it’s like flow chart medicine and that…takes away any thought process from it…I think for some patients, that just not appropriate to do that.” *(Orthopaedic Consultant D)*
		“You’re just following a flow chart; they’re complex patients and it takes out some of the thought process.” *(Orthopaedic Consultant G)*
	- Poor dissemination of guidelines - Limited education	“I don’t think there was a lot of fanfare made of that…and I don’t know how many changes were involved in those new guidelines, but certainly I haven’t come across significant changes communicated to me by the surgeons. And I would have hoped that they would be a little bit aware of what those guidelines particularly pertain to with respect to the procedures they’re doing.” *(Anaesthetist E)*
		“Those PROMPT update emails are very text heavy and it’s a lot of stuff that’s not relevant. It’s actually hard to find anything relevant specifically to us within those if we got a chance to look at it with the e-mail fatigue that we have as well.” *(Anaesthetist G)*
		“We also have a FORMS [PROMPT] protocol update email that comes through regularly. So, it would be amongst those. But look, it would be one change in a protocol amongst many others, and so easily missed unless you’ve got a special interest in it. You wouldn’t look at it, I think.” *(Pharmacist C)*
		“I know at some point last year that was what was being done [single dose prophylaxis]. And then the problem is the nurses weren’t told about this. Then there were many, many questions from the nurses when the antibiotics were not charted…So it needs to be a whole department level and this information needs to be also distributed to the nurses that we are not doing [postoperative] antibiotics anymore for closed fracture ORIF.” *(Orthopaedic Registrar D)*
Social influences	Little engagement with stakeholders	“The biggest thing I would have thought, with any sort of guidelines like this would have come up by the Infectious Disease department and they would have done something like what you have if they were smart – is target the people who are giving these antibiotics, which is us, and they should have come and talk to us and say, ‘Hey, these are new guidelines, this is where you find it and this is the summary of what it is.’ And that would be the perfect way to target the correct audience, I think, with targeted information.” *(Anaesthetist B)*
		“They don’t understand the surgeon’s perspective of these. So, there is a surgeon and physician role in these. Certainly, when antibiotics are being prescribed, I think when guidelines are being made, I think surgeons should be involved in it.” *(Orthopaedic Consultant H)*
		“Guidelines are a broad brush that’s written not by a surgeon generally. And you might choose that you want greater coverage than what is suggested as ok in that circumstance.” *(Orthopaedic Consultant D)*
**TDF Domains**	**Enablers**	**Illustrative quotes**
- Environmental context and resources - Behavioural regulation - Reinforcement - Knowledge	- Presence of local guidelines - Greater accessibility - Enhanced dissemination of guidelines	“I agree that local guidelines are important because there’s so many other guidelines only to vary by a little bit to completely confuse everybody. So, I think any guidelines have to be local, but they all have to be stuck on the wall right in front of us. Otherwise, they’re unlikely to get followed, given that there are conflicting guidelines.” *(Anaesthetist F)*
		“I have used a laminated decision-making tool at a different hospital before and found that very helpful to select the right antibiotic and I think a lot of my current practice, in addition to consulting with the surgeons, actually goes back to having used one of those flowcharts and having a lot of that just burned into my brain actually.” *(Anaesthetist D)*
	Improving guideline specificity	“My suggestion of if there is a surgical based guideline rather than a medical based guideline…if the guideline is exhaustive, then you have all scenarios and permutations and combinations, it’s easy for the surgeon to use.” (*Orthopaedic Consultant H)*
		“I think it would be beneficial to have more specific examples in the guidelines. You know, for arguments sake, if you had patients who were immunocompromised with these conditions, you should consider this even if it’s no change in standard practice. You know an answer like that is still an answer…Or if you’re doing revision fixation for a fracture, this is what you should do. And again, even if at the end of the day, it’s always still the same antibiotics, at least then there is more specificity for the particular case you might be looking up.” *(Orthopaedic Registrar E)*
	Using technology to improve practice	“I quite like the idea of an app…or at least an online type decision making tool that can look like an app when you open it up. And I think that that’s probably easier to update than paper guidelines.” *(Anaesthetist A)*
		“Maybe even like a QR code where they could just link – go into it straight away using their phones. Something very accessible, I’d say.” *(Pharmacist A)*
	Education	“People need to be made aware of its existence early on in a way that they’re actually gonna [sic] take that on board… And it needs to be done effectively when staff changeover ‘cause one of the problems with changeover of staff is you get lumped with about 10,000 documents the day you arrive and some things are more important than others, and I’d say this is one of the more important things. So, if it can have its own orientation when new staff arrives, they’re aware of the policy, protocol, and they actually take that on board.” *(Orthopaedic Registrar A)*
		“Presentation at the start [during orientation] would be the best when you’re just listening. And I find handouts…I’m less likely to review.” *(Orthopaedic Registrar C)*
		“I think what we need is an education campaign on changes to our in-house prescribing guides because a lot of people wouldn’t be familiar with them and so a refresher doesn’t hurt. I think even if we could talk at a Grand Round or… just get the word out there more officially I think rather than each individual pharmacist sort of tapping the doctor on the shoulder. And all of us being on the same page too.” *(Pharmacist C)*
Social influences	Stakeholder engagement	“I think if we are trying to set up a protocol…speaking to the head of consultants as well the consultants of the surgical teams and actually finding out what their processes and what their preference is…because they are the one at the end of the day [that] dictates what treatment the patient gets. So, speaking to them, getting a guideline and also finding where their references are. And then from that forming a protocol or policy around it and then doing the education.” *(Pharmacist E)*
		“It’s challenging because the way to introduce it will really be a national level like AOA [Australian Orthopaedic Association] level of conference, but it’s not something that everybody will pick up readily at the same time. So… then constant reminder is always necessary until you get organisational level shift in paradigm.” *(Orthopaedic Registrar D)*

*“There’s always a conflict between physicians and surgeons... ID [infectious disease] physicians, general physicians – when they suggest something, we always have to take our surgical perspective into account.”* (Orthopaedic Consultant H)

#### 2.2. Guideline accessibility.

Accessibility and availability of guidelines were viewed with different perspectives, with some participants stating that local (in-house) guidelines were easily accessible,

*“It’s just on PROMPT and it’s just a click away. It’s also within an intranet page as well if they actually search up antimicrobial stewardship.”* (Pharmacist D)

whilst others found it cumbersome to access both national and local guidelines stating that *“they’ve been useless to find”* (Orthopaedic Registrar C) (S3 Table).

Participants also noted that accessing guidelines could be limited due to the unavailability of physical resources.

*“IT access as well. For example, if all the computers in theatre are being used by nurses or somebody else that you don’t actually have access on the spot to search.”* (Orthopaedic Registrar B)

#### 2.3. Documentation.

Administering SAP prior to incision is crucial to prevent surgical site infections [8], with the correct timing of administration used as a key indicator to assess the appropriateness of SAP [5,30]. However, participants reported that incision time is often not documented, ultimately making it difficult to assess whether antibiotics are administered at the appropriate time in line with guideline recommendations.

*“We don’t document that… Some of us will mark the anaesthetic chart as to when surgery starts. But that’s not a routine, and we’re not asked to do that. So, I doubt whether anybody’s recording it.”* (Anaesthetist A)

Participants also noted that it was difficult to ascertain whether vancomycin was necessary as an additional antibiotic due to poor documentation.

*“Sometimes not actually even clearly documenting why they want it [vancomycin]. You sometimes have to do a bit of reading to work out why. Plus, a little bit of a question to the intern.”* (Pharmacist D)

Furthermore, the reasons for why a patient may require an extended duration of SAP is not clearly communicated in the postoperative setting.

*“It’s just charted usually, and it will be… maybe IV antibiotics for 72 hours, but the reason, it’s not usually put down.”* (Orthopaedic Registrar D)

#### 2.4. Environmental or surgery related factors influencing prescribing practice.

Several factors were reported by participants as influencing the choice, timing, and duration of antibiotic administration for ORIF procedures.

**2.4.1. *Workflow influencing choice and timing of antibiotic administration*:** Current Australian guidelines (*Therapeutic Guidelines: Antibiotic* v16*)* recommend the use of vancomycin (instead of clindamycin) for ORIF procedures in patients with a severe penicillin allergy [4]. The decision to administer clindamycin over vancomycin can be impacted by workflow and communication between the surgical and anaesthetics team (S3 Table).

*“One of the barriers to the use of vancomycin is the fact that it needs to be infused over an hour. And so, that would be part of the decision making, particularly if it was a very short procedure.”* (Anaesthetist A)

Whether or not antibiotics are administered at the correct time in relation to incision timing is also influenced by *“how long it takes to position”* a patient in theatre and can vary from “*5 minutes or so”* or “*be much longer”* (Anaesthetist A).

**2.4.2. *Duration of inpatient stay*:** How long a patient stays in hospital both prior to and after surgery can determine the type of antibiotics administered as well as whether postoperative doses are prescribed (S3 Table).

*“If they’ve been an inpatient for a period of time, the surgeons might have a particular view on what they want… depending on swab results and what’s grown and the like.”* (Anaesthetist B)

*“To be honest about it, if they’re gonna [sic] go home that day, then they’ll only get a single dose.”* (Orthopaedic Consultant D)

**2.4.3. *Duration of surgery or complexity of fracture*:** The administration of single dose prophylaxis can also be determined by “*the complexity of the fracture and the time required…to fix the fracture,”* with single dose prophylaxis more likely to be administered for *“straightforward”* procedures (Orthopaedic Consultant H).

**2.4.4. *Private vs public sector*:** Orthopaedic consultants also commented on how their practice varied based on whether they were working in a public or private hospital, with greater opportunities to influence SAP decision making in the private setting.

*“The concern is in a public system I may not have the same influence on my patients like that in a private system. The private system is my patient. I know what the patient is like, I have a control on things. In the public system, sometimes you don’t have that.”* (Orthopaedic Consultant H)

In terms of guideline access in a public hospital setting, orthopaedic consultants reported they would “*use”* their *“local registrar”* to provide guideline information as registrars are often *“up with the latest”* (Orthopaedic Consultant F).

In a private setting, however, orthopaedic consultants stated they would often contact an infectious disease (ID) physician for advice.

*“In a private setting, I’ll often ring an ID consultant and say ‘Hey I’ve got this. What do you think?’ And then we’ll talk about it together.”* (Orthopaedic Consultant D)

### Domains 3 and 4: ‘Skills’ (3) and ‘Beliefs about capabilities’ (4)

#### 3.1. Autonomy and variability in practice.

Participants also spoke of the inevitable “*heterogeneity in practice,*” despite the “*evidence*” that may exist, with *“anecdotal evidence [having] a role to play”* in practice (Orthopaedic Registrar E). Orthopaedic consultants also discussed the need to practice as they see fit, particularly if a patient did not fit within the mould of standard guidelines (S3 Table).

*“Unless it’s mandatory, there’s gonna [sic] be variations and we should respect those variations. I feel as a consultant we have a right to choose the treatment unless it is proven, and it is decided by a governing body or a committee, that this is a better treatment.”* (Orthopaedic Consultant K)

#### 3.2. Experience.

The number of years practised as well as both positive and negative incidents experienced during one’s career were also reported as determinants of practice (S3 Table).

#### 4.1. Habits developed during practice and communication between staff.

Standard or routine practice will often dictate how antibiotics are prescribed, which can result in limited communication about postoperative antibiotics (S3 Table). For example, for ORIF procedures *“the standard expectation is three doses of antibiotics, so one preoperatively and then two postoperatively”* (Orthopaedic Registrar E).

Participants also reported on how anecdotal evidence will be used in preference to evidence-based guidelines.

*“You can have very clear evidence for things, and everyone’s got different opinions on it, and anecdotal preference will always reign.”* (Orthopaedic Registrar E)

#### 4.2. Situations in which guidelines may be used to guide practice.

When asked about which type of guideline would be accessed if necessary, almost all participants reported they would use local guidelines and *“very much prefer local as opposed to international guidelines because that would be geared towards the bacteria that is prevalent”* (Orthopaedic Consultant E) within the hospital.

Orthopaedic consultants also stated that guidelines are *“an excellent tool to guide junior staff”* (Orthopaedic Consultant E) and assist *“in terms of making a quick streamlined decision for standard cases”* (Orthopaedic Consultant C).

Multiple participants mentioned they would also use local guidelines to determine the choice of antibiotic in situations where patients had *“an allergy to the known antibiotic”* (Orthopaedic Consultant J), in *“contaminated or farmyard type injuries,”* (Orthopaedic Consultant D) or if there was a *“co-existing infection…and the organism is shown to be resistant to any antibiotics”* (Orthopaedic Consultant I).

### Domains 5 and 6: ‘Social/professional role and identity’ (5) and ‘Social influences’ (6)

#### 5.1. Roles and responsibilities regarding SAP decision making.

Participants were asked to describe their roles when it came to dealing with SAP for ORIF procedures. Orthopaedic “*consultants or surgeons are the ones who drive”* (Orthopaedic Consultant J) SAP decision making, with the anaesthetist often *“asked to administer antibiotics as prophylaxis for infection…at the request of the surgical team”* (Anaesthetist A). However, antibiotics were not viewed as *“an anaesthetic drug in a lot of anaesthetist’s eyes”* but rather *“a surgical drug”* (Anaesthetist F). The administration of intraoperative antibiotics was often done *“automatically”* by the anaesthetics team (Anaesthetist D).

Orthopaedic registrars are also involved in SAP decision making, by *“prescrib[ing] antibiotics either empirically or therapeutically depending on the initial diagnosis,”* (Orthopaedic Registrar A), with the final decision made by the orthopaedic consultant (S3 Table). Postoperatively, little communication about antibiotic orders may take place, such that *“if nothing is mentioned”*, registrars *“assume that [the consultants] want postop antibiotics”,* with registrars stating *“they haven’t run into issues”* by making this assumption (Orthopaedic Registrar D).

Pharmacists perceived their role to include a *“thorough medication history”* as well as to review “*pre-surgery antibiotics”* and ensure antibiotics are *“cease[d] after surgery”* (Pharmacist A).

#### 6.1. Hierarchy and relationship with senior staff.

Orthopaedic registrars mentioned that although they may be aware of guidelines, it was unlikely to be followed if their seniors were not aware.

*“We will play second fiddle to whichever treating consultant is on and if they’re not aware of the guideline, then that’s the biggest barrier to using it or following it.”* (Orthopaedic Registrar A)

Junior surgeons also reported on how the decision of orthopaedic consultants will ultimately influence their practice (S3 Table).

*“I can completely agree with your presentation [regarding guideline content] but my opinion is worthless if whether or not my consultant agrees. And if my consultant doesn’t agree, then I’ll continue prescribing [postoperative antibiotics].”* (Orthopaedic Registrar D)

Pharmacists also reported on how hierarchy influences practice, with the need for consultants to agree with recommendations before an improvement in practice can be observed.

*“It has to come from the top down as well, because they can argue as much as they like that antis [antibiotics] aren’t indicated, but then if the consultant says ‘No, I want them’, yeah, that’s it. Nothing’s gonna [sic] change.”* (Pharmacist C)

#### 6.2. Relationship with ID physicians.

As discussed previously, collaboration with an ID physician was viewed positively by orthopaedic consultants, with consultants mentioning they would *“be speaking with the infectious diseases team for advice rather than just looking up a standard guideline”* (Orthopaedic Consultant I)*.*

Participants also spoke of how they would *“kindly utilise the infectious disease team as a scapegoat and say that they recommend something different”* in situations where antibiotics prescribed are *“categorically wrong…for a particular class of bacteria”* (Orthopaedic Registrar E).

Pharmacists also noted they would often consult with ID if they could not identify a *“clear indication for a medication”* or “*if there’s a bit of reluctance from the doctors to make changes”* (Pharmacist C)*.*

### Domains 7, 8 and 9: ‘Emotion’ (7), ‘Belief about consequences’ (8) and ‘Optimism’ (9)

#### 7.1. Fear.

The concept of fear, particularly in relation to infection development (i.e., surgical site infections), was heavily discussed throughout the interviews by all groups of participants. Fear was identified as a factor that greatly influences the decision to prescribe postoperative antibiotics, thereby driving the overuse of antibiotics and deviance from current guideline recommendations (S3 Table).

*“I guess if you’re a surgeon and you’re confronted with the patient that has a surgical site infection, that will cloud your judgement.”* (Anaesthetist C)

Orthopaedic consultants also admitted that although the risk of infection remains *“low”,* the consequences can be *“disastrous”* (Orthopaedic Consultant I) if an infection develops, thus leading to additional doses being prescribed.

This concern is particularly evident in situations where patients may be physiologically unwell, or in situations where the risk of infection is greater.

*“If you’re dealing with someone who’s physiologically not the greatest, and you’re doing big revision surgery, you really don’t want infection, like really don’t want infection.”* (Orthopaedic Consultant D)

There is also the fear of litigation, whereby the surgeon’s career is at risk if a patient comes to harm.

*“The other thing is when there is a guideline and you deviate from it, you are doing it at your own risk. Or something goes wrong or doesn’t work, then there is always a pointing finger or there could be issues of litigation that you didn’t follow the guidelines.”* (Orthopaedic Consultant H)

#### 8.1. Why change practice if the current one is effective and does not cause harm?.

The consequences of using additional postoperative doses were not seen as harmful in light of antimicrobial resistance, but rather as something that could reduce the risk of harm with very little side effects (S3 Table).

Participants also noted there is little need to change practice, particularly when an effective and non-harmful regimen is currently being employed.

*“If what we’re currently doing is not causing harm, then it’s very hard to change our current practice, even though there’s evidence out there saying that there is an alternative. It’s like the saying ‘why fix it if it’s not broken’ sort of thing.”* (Orthopaedic Consultant I)

#### 9.1. Optimistic attitudes towards single dose prophylaxis.

Nonetheless, despite attitudes towards postoperative antibiotic administration, participants explained there is a possibility for single dose prophylaxis to be implemented in the future, particularly if this concept is *“unanimously”* accepted (Orthopaedic Consultant H) (S3 Table).

*“I think there is certainly a role for day surgery coming up. And with the current guidelines, if you got a simple ORIF for example, let’s say a hand or a foot fracture, you operate and they go home. I guess many times we tend to use just single dose.”* (Orthopaedic Consultant K)

### Domains 10, 11 and 12: ‘Memory, attention and decision processes’ (10), ‘Behavioural regulation’ (11) and ‘Reinforcement’ (12)

#### 10.1. Memory.

Participants discussed how postoperative antibiotics would often continue to be administered *“as the doctor will forget to stop them after 24 hours”* (Pharmacist A) (S3 Table).

One orthopaedic consultant also mentioned that *“unless it comes up”* or they are asked *“specifically*”, they would often forget to inform registrars that their *“policy is just the one”* dose of antibiotics (Orthopaedic Consultant B).

Without effective dissemination of guidelines, participants also reported on how *“it’s kind of relying on someone to feel like reading PROMPT or to remember the guidelines, particular surgical type, particular hospital you are [at] on the day”* which is *“doomed to failure.”* (Anaesthetist F).

#### 10.2. Additional factors that influence decision making.

Although there are various factors that influence SAP decision making, simply put it is a combination of *“experience, fear and superstition”* (Orthopaedic Consultant F).

Patient factors, as well as limb fracture site, also heavily influences the decision to prescribe postoperative doses (S3 Table).

*“If you’re immunocompromised, diabetic, smoker, if you have particularly a lower limb injury, particularly a foot and ankle incision, then I would keep you and give you the two doses after rather than send you home on the day.”* (Orthopaedic Consultant E)

#### 11.1. Proposed enablers to changing practice.

To increase guideline use, participants recommended that a *“multimodal approach”* be used, for example *“sending out an email”* informing of guideline updates, disseminating guidelines through the *“weekly newsletter”*, *“weekly meetings”* and during *“consultant clinic”* (Orthopaedic Registrar E).

Simplifying access to guidelines was also suggested by keeping guidelines within reach (Table 2):

*“We can add a page with summarised guidelines to that [anaesthetic record sheet] so that when they’re filling in all that paperwork before doing surgery, it’s right there in front of them and that will prompt them to look at it and then prescribe the accurate [antibiotic].”* (Pharmacist A)

Posters displayed in a frequently used location was also suggested.

*“The other thing is maybe have a simple grid printed out and stuck to a place where we usually write op [operation] reports, so in theatre.”* (Orthopaedic Registrar B)

It is important, however, that posters are not displayed in an area where there is already the heavy use of other posters.

*“There’s so many policies, it’s really good, but when you see this wall of crap, you don’t really take the time to look at it.”* (Orthopaedic Registrar A)

#### 11.2. Audit and feedback.

Audits were viewed as a beneficial process and a means of improving practice (S3 Table).

*“That’s useful for myself, to see where my practice might differ and if I’m an outlier, it’s gonna [sic] make me question whether I’m doing the right thing, whether I need to review what I’m doing.”* (Orthopaedic Consultant B)

However, some believed audits would make a difference *“only if it affects [the] consultants”* (Orthopaedic Registrar D), highlighting once more the importance of stakeholders, particularly the consultants, agreeing with guideline recommendations as they often make the final decision.

#### 12.1. Reminders and reinforcements.

The concept of repetition and *“regularly reinforcing”* information regarding SAP guidelines, particularly *“at the start of rotations would be helpful”* (Pharmacist C) in modifying practice (S3 Table).

*“I think realistically the best form of delivery of information like this is through repetition and forced exposure so to speak.”* (Orthopaedic Registrar E)

## Discussion

This study has demonstrated that a complex interaction exists between the factors that influence SAP decision making in orthopaedic surgery, with a combination of factors ultimately impacting the extent of guideline adherence. These factors include inter- and intrapersonal factors, such as knowledge, habits and hierarchical relationships, as well as factors that pertain to the local environment such as workflow and accessibility of guidelines.

The Surgical National Antimicrobial Prescribing Survey (Surgical NAPS) is a standardised tool used in Australian hospitals to assess the appropriateness of procedural and postprocedural antibiotic prescriptions [2]. Recent findings from the Surgical NAPS have identified multiple areas of improvement in orthopaedic surgery, particularly in relation to the administration of SAP at the correct time and for the correct duration [2].

The correct timing of SAP administration can be impacted by a range of factors. SAP administration in theatre has previously been identified as ‘low priority’ for surgeons and anaesthetists alike, especially in complex situations or during lengthy procedures [14,15,17,31]. This can result in preoperative doses being administered at a suboptimal time and intraoperative doses being omitted. Furthermore, workflow can also influence when SAP is administered in relation to incision timing [17,32]. As stated by one anaesthetist in this study, the time taken to position a patient in theatre can determine when the preoperative dose is administered. Another key finding from the Surgical NAPS is the poor documentation of incision timing (only present in 66.1% of cases audited) [2]. As noted by one participant, there is no requirement to record incision timing at our hospital, thus, an assessment of the appropriateness of timing cannot be made. The administration of SAP at the correct time is a key quality indicator used to assess appropriateness of SAP [5,30], hence interventions targeting this can be designed to improve practice.

From the results of this study, it is clear that the responsibility of SAP lies with the surgeon, a finding that is reinforced in the literature [14,16,17,19,21,32]. All orthopaedic consultants agreed that SAP decision making is consultant driven, with input provided by the orthopaedic registrars, particularly in the public hospital system. However, on the wards, the responsibility of charting postoperative antibiotics lies with the junior surgical team (registrars, residents etc.), thus the importance of clearly communicating postoperative antibiotic plans [14,19,31]. Without clear communication, postoperative antibiotics can continue to be prescribed unnecessarily, contributing to the inappropriate use of antibiotics. Of interest, anaesthetists discussed how they did not perceive antibiotics to be an “anaesthetic drug” but rather a “surgical drug”, with their role limited to administering the antibiotic in theatre. This is similarly reported by Tan et al. [17] in which anaesthetists expressed resentment over having to administer antibiotics, a task they considered outside their main scope of practice.

To ensure that SAP is prescribed and administered appropriately in line with guideline recommendations, it is vital that the roles and responsibilities of all healthcare professionals involved are clearly defined, whilst recognising the prevalent hierarchical relationships present in the surgical setting. In this study, orthopaedic registrars commented on how their practice is influenced by their consultant’s prescribing behaviours such that they will continue to follow consultant practice despite awareness of SAP guideline recommendations. This power imbalance may result in the continuation of practices that prevent the judicious use of antibiotics [21].

Thus, as it is the orthopaedic consultants who lie at the top of the metaphorical ‘food chain’, it is crucial that they are involved in the development and dissemination of SAP guidelines as guidelines are less likely to be adhered to without consultant agreement of recommendations [18]. The involvement of orthopaedic surgeons in the design process of guidelines (alongside other measures such as education) has been shown to increase the uptake of guidelines [33]. In a study by Lohiniva et al. [34] orthopaedic surgeons involved in the development of a behaviour change intervention have suggested that consensus of guideline recommendations should be reached first by senior staff during the development phase of guidelines, followed by its gradual introduction through training and orientation. Where barriers to the use of SAP guidelines have been identified, senior ownership and engagement of intervention strategies have been positively associated with an increased compliance to guideline recommendations [35]. Although senior orthopaedic surgeons were involved in the development of local SAP guidelines with consensus reached, the lack of specific, tailored recommendations or effective dissemination strategies may have impeded their uptake in clinical practice. A more nuanced approach, which considers the complexity of healthcare delivery and the unique circumstances of different settings, would enhance the likelihood of successful implementation.

In this study, multiple orthopaedic consultants reported on the broadness of SAP guidelines as being a major barrier to its implementation in the clinical setting. Throughout the interviews, it was evident that duration of SAP (i.e., whether a patient receives single dose or multi-dose prophylaxis) was linked to patient and procedural factors. Patient heterogeneity, physiological status, limb fracture sites as well as non-ideal theatre environments, can all influence the risk of infection, with these reasons stated by orthopaedic consultants and registrars as reasons for the administration of postoperative antibiotics. These nuances are not taken into account in current guidelines. For guidelines to be accepted, key users of such guidelines must find it applicable to their patients. As posed by participants in this study, tailoring guidelines to include more detailed scenarios for how SAP should be prescribed in different situations can assist with the gradual transition to the use of single dose prophylaxis for ORIF procedures.

It is known that overcoming the inertia of habitual practice is difficult [36,37] as social norms or culture can heavily influence practice patterns and adherence to guidelines. Thus, when considering implementation strategies to increase the uptake of SAP guidelines in this setting, the impacts of the factors listed above should be thoroughly considered. The use of a theory to inform targeted intervention or implementation strategies can improve the robustness of a study [38]. Likewise, frameworks such as the TDF [21] and the Action, Actor, Context, Target, Time (AACTT) framework [39] can be used to assist in the design of interventions that target guideline adherence by understanding the behaviours that lead to practice change. It is unlikely that the implementation of a single strategy is sufficient to modify practice, with the targeting of multiple factors likely to be required. Constant education and repetition are needed, particularly during staff changeover periods, to increase the uptake of guideline recommendations.

Strengths of this study include the use of a validated framework to design the interview questions and analyse results. Insights are gathered from a range of key stakeholders involved in SAP decision making with variable lengths of experience, which allows for a wider range of perceptions to be explored. Of the limitations, we were unable to interview nursing staff. Ward nurses play a vital role in championing the safe and effective administration of medication to patients. Their perceptions of their responsibilities for dealing with SAP, views on single dose prophylaxis and relationships with surgical teams can provide valuable insight into understanding what influences the administration of postoperative antibiotics. Furthermore, as this study explores the prescribing practices of healthcare professionals, there is the potential for certain details to be omitted due to social desirability bias. This study also comprises of a small cohort of healthcare professionals at one tertiary hospital in Australia. Although the barriers to implementing SAP guidelines will differ at each hospital, we believe that the results of this study are transferrable to other settings as similar concepts regarding the complexities of SAP decision making have been reported in the literature.

## Conclusion

A multitude of factors influence the way SAP is prescribed for ORIF procedures, with many barriers (both personal and environmental) impacting guideline adherence. By understanding these factors and the local context, targeted interventions can be designed to improve the use of SAP guidelines, ultimately taking us one step closer to shortening the evidence-practice gap. By implementing recommendations in current SAP guidelines, the excessive and inappropriate use of antibiotics can be reduced, thereby reducing the burden of antimicrobial resistance in the long term.

## Supporting information

S1 Table Interview guide for stakeholders. (PDF)

S2 Table COREQ checklist. (PDF)

S3 Table Quotes illustrating the 12 domains of the TDF. (PDF)
