# Supplementary material for: User perceptions of surgical antimicrobial prophylaxis guidelines in orthopaedic surgery in a tertiary Australian hospital
Source: PLoS One. 2025 Mar 20;20(3):e0319829. doi: 10.1371/journal.pone.0319829 (PMC11925292; doi:10.1371/journal.pone.0319829)
Supplement: S1 Table — (PDF) [file pone.0319829.s001.pdf]

S1 Table. Interview guide for stakeholders

**Questions for anaesthetists**	
**TDF domain**	**Questions**
Social/professional role and identity	Using ORIF of closed fractures as an example for the discussion:<br><br>Can you tell me a little bit about your role when it comes to managing antibiotics for ORIF patients?
Skills/ Beliefs about capabilities	When would you consult the guidelines?
Environmental context and resources	Is it sometimes difficult to follow SAP guidelines? Can you tell me a little bit about why?<br>*(Prompt: What are problems with current guidelines?)*<br><br>Can you tell me about what makes it easier to follow SAP guidelines?<br>*(Prompt: How would you improve guidelines?)*
Knowledge	How do you find out about new guidelines?<br><br>Do you think the presence of local guidelines has affected your practice? If so, how?
Social influences	Can you tell me about how you talk to your specialist colleagues about changes to prophylactic antibiotic use?<br>*(Prompt for SH: How do you manage situations where you and your colleagues do not agree with antibiotic decisions?)*
Behavioural regulation	The antibiotic stewardship team provide data from audits they undertake, can you tell me about your opinions regarding this process?
Conclusion	Are there any further comments or experiences you would like to share?
**Questions for orthopaedic consultants**	
**TDF domain**	**Questions**
Social/professional role and identity	Using ORIF of closed fractures as an example for the discussion:<br><br>Can you tell me a little bit about your role when it comes to prescribing antibiotics in surgery?
Skills/ Beliefs about capabilities	When considering antibiotics for these patients, what influences your decision making?<br>*(Prompt: which patients are more likely to receive single dose prophylaxis vs postoperative doses?)*<br><br>When would you consult the guidelines?
Environmental context and resources	Is it sometimes difficult to follow SAP guidelines? Can you tell me a little bit about why?<br>*(Prompt: What are problems with current guidelines?)*<br><br>Can you tell me about what makes it easier to follow SAP guidelines?<br>*(Prompt: How would you improve guidelines?)*
Knowledge	How do you find out about new guidelines?<br><br>Do you think the presence of local guidelines has affected your practice? If so, how?
Social influences	Can you tell me about how you talk to your specialist colleagues about changes to prophylactic antibiotic use?<br>*(Prompt: How do you manage situations where you and your colleagues do not agree with antibiotic decisions?)*
Behavioural regulation	The antibiotic stewardship team provide data from audits they undertake, can you tell me about your opinions regarding this process?
Conclusion	Are there any further comments or experiences you would like to share?

Questions for orthopaedic registrars	
**TDF domain**	**Questions**
Social/professional role and identity	Using ORIF of closed fractures as an example for the discussion:<br><br>Can you tell me a little bit about your role when it comes to prescribing antibiotics in surgery?
Skills/ Beliefs about capabilities	When considering antibiotics for these patients, what influences your decision making?<br>*(Prompt: which patients are more likely to receive single dose prophylaxis vs postoperative doses?)*<br><br>When would you consult the guidelines?
Environmental context and resources	Is it sometimes difficult to follow SAP guidelines? Can you tell me a little bit about why?<br>*(Prompt: What are problems with current guidelines?)*<br><br>Can you tell me about what makes it easier to follow SAP guidelines?<br>*(Prompt: How would you improve guidelines?)*
Knowledge	How do you find out about new guidelines?<br><br>Do you think the presence of local guidelines has affected your practice? If so, how?
Social influences	Can you tell me about how you talk to your senior colleagues about potential changes to prophylactic antibiotic use?
Behavioural regulation	The antibiotic stewardship team provide data from audits they undertake, can you tell me about your opinions regarding this process?
Conclusion	Are there any further comments or experiences you would like to share?
**Questions for pharmacists**	
**TDF domain**	**Questions**
Social/professional role and identity	Using ORIF of closed fractures as an example for the discussion:<br><br>Can you tell me a little bit about your role when it comes to reviewing patients who have undergone or are undergoing surgery?
Skills/ Beliefs about capabilities	When would you consult the guidelines?
Environmental context and resources	Is it sometimes difficult to follow SAP guidelines? Can you tell me a little bit about why?<br>*(Prompt: What are problems with current guidelines?)*<br><br>Can you tell me about what makes it easier to follow SAP guidelines?<br>*(Prompt: How would you improve guidelines?)*
Knowledge	How do you find out about new guidelines?<br><br>Do you think the presence of local guidelines has affected your practice? If so, how?
Social influences	Can you tell me about how you talk to your specialist colleagues about changes to prophylactic antibiotic use?<br>*(Prompt: How do you manage situations where you and your colleagues don't agree with antibiotic decisions?)*
Behavioural regulation	The antibiotic stewardship team provide data from audits they undertake, can you tell me about your opinions regarding this process?
Conclusion	Are there any further comments or experiences you would like to share?
