# Supplementary material for: User perceptions of surgical antimicrobial prophylaxis guidelines in orthopaedic surgery in a tertiary Australian hospital
Source: PLoS One. 2025 Mar 20;20(3):e0319829. doi: 10.1371/journal.pone.0319829 (PMC11925292; doi:10.1371/journal.pone.0319829)
Supplement: S3 Table — (PDF) [file pone.0319829.s003.pdf]

S3 Table. Quotes illustrating the 12 domains of the TDF

Quotes illustrating the domains 'Knowledge' (1) and 'Environmental context and resources' (2)		
**TDF domains**	**Key concepts**	**Illustrative quotes**
Knowledge	Lack of awareness of guideline recommendation	"I realised that although there is a change in protocol, it's not actually followed… because I once brought it up with a doctor and they were just like 'Oh but it is standard practice for us to just give it.' So, I don't think people are aware of our change in protocol." *(Pharmacist E)*
		"Thing that I've found is that often when you're made aware of a guideline, not all of the surgeons as part of that surgical craft group are even aware of the change…often I think I've brought up to the surgeon did they want 3 grams [of cefazolin] because the patient's greater than 120 [kg] and they weren't aware of that change. So, the information doesn't seem to be widely disseminated, or perhaps widely agreed by all working in that area." *(Anaesthetist C)*
	Awareness of guideline recommendation	"I mean there is evolving evidence that a single preop antibiotic is sufficient for standard fracture with the patient with no risk factor. However, I haven't I'm afraid quite changed, so I go with one preop, two postop." *(Orthopaedic Consultant C)*
		"Oh, I mean, my understanding is that the evidence supports just a single dose antibiotic at induction for closed fracture ORIFs and even for joint replacement." *(Orthopaedic Registrar D)*
		"The evidence suggests that administration of a single preoperative dose of antibiotics is adequate. And I don't believe that there's much evidence for or against two further doses. I know there's not much evidence to support any continuing antibiotics outside of that first 24-hour period." *(Orthopaedic Registrar E)*
	Importance of agreement with guideline recommendations	"Just an understanding of where the guidelines have come from, the evidence for it and then people's agreement to adopt the guidelines." *(Anaesthetist C)*

		“Coming to a consensus with the head [of unit] because the junior doctors often do listen to us, but they’re also often very scared to change therapies because the consultant has said so.” *(Pharmacist E)*
Environmental context and resources	Accessibility	“Accessibility is an issue. So, we have Therapeutic Guidelines available via the portal at the hospital. To get to that, we need to do at least five or six clicks… in terms of its availability in theatre, I still haven't seen one poster on the wall.” *(Orthopaedic Consultant E)*
		“I think accessibility is a big deal, especially if you've got something like a surgical antibiotic guideline that’s different for every different type of surgery.” *(Anaesthetist F)*
	Workflow/Logistics<br><br>Communication	“The time that we have these discussions is often just at the time of induction or very close to the time of induction. So, getting clindamycin from our fridge seems to be a lot quicker than actually getting the vancomycin and actually trying to get the plasma level up before the tourniquet goes up.” *(Anaesthetist D)*
		“I still prefer to give three doses. If they leave the hospital before the third dose is given, I don't mind too much, but still prefer to have three doses rather one.” *(Orthopaedic Consultant F)*
		“I think we do too many cases [that are] overnight stay anyway, and then by convention they get antibiotics.” *(Orthopaedic Consultant G)*
	Environmental factors influencing prescribing	“The environment of the surgery is not ideal. You have foot traffic going through the theatre, you can never guarantee sterility when the scrub nurses open implants, especially if you're doing these trauma cases in the middle of the night. It's not a controlled environment. The patients are not controlled patients.” *(Orthopaedic Consultant I)*
		“Patients being in a hospital for a while, some people will have thought about using vancomycin just as a prevention for MRSA infections. And yeah, surgery factors and how long the operation went…whether you felt that that operation was associated with high risk of infection such as like foot and ankle surgery.” *(Orthopaedic Registrar B)*

Quotes illustrating the domains 'Skills' (3) and 'Beliefs about capabilities' (4)		
**TDF domains**	**Key concepts**	**Illustrative quotes**
Skills	Autonomy<br><br>Variability in practice	"We tend to have a policy of one dose intraoperatively [induction dose], but we have discretion to give more than one. So, some of us follow the unit policy and some of us decide on additional doses afterwards." *(Orthopaedic Consultant A)*
		"These are the cases that don't follow guidelines because they're complex cases where deviation should be allowed. These are the ones where we need to be given some leeway as physicians and surgeons to apply the latest to complex cases rather than being limited by broad and local guidelines." *(Orthopaedic Consultant E)*
	Experience	"It's a combination. Journal clubs, readings, conferences as well as the experience from my own complications or negative outcomes. It also shapes your practice, and you certainly change your decision making if one thing is working for you versus the other." *(Orthopaedic Consultant K)*
		"I think the main thing is the interns hesitate to change anything without discussing it with their reg [registrar]. They always wait until they get that final ok, even if it's on the guideline or even if it's seen by an AMS [antimicrobial stewardship] pharmacist." *(Pharmacist A)*
Beliefs about capabilities	Habits<br><br>Communication	"If you're encountering practice that hasn't changed, it's because we just do the same thing unless someone tells us to do otherwise." *(Anaesthetist F)*
		"Generally, I've found in my practice that people will still prescribe ongoing for 24 hours because it's convention, even though there's no evidence for it, and sometimes it'll treat me if I'm worried that patient's got particularly high-risk factors such as poor skin, poor hygiene or physiological status." *(Orthopaedic Consultant D)*
		"It's one of those things where we've been following this sort of set protocol for a while and with seemingly reasonable results." *(Orthopaedic Consultant J)*

		"I feel like they are very set in their ways of giving the antibiotics. I don't know whether any other pharmacists have seen that because I have seen it and they refused to still cancel the antibiotics post discussion because it's what they've always done." *(Pharmacist E)*
	Communication and relationship with seniors<br><br>Self-confidence	"The first part is communication and access. We don't have a problem with that. We can get onto them [consultants] and get an answer easily. The problem is more really the crux of the issue, which is if we're following a guideline, or they're just sort of making their own clinical judgements not necessarily based on anything good. Then we may have trouble if we're defining that as us trying to follow the advice of the system and then going against it. Actually getting a decision off them is easy. Whether that decision is what a policy may say or not is the harder part." *(Orthopaedic Registrar A)*
		"They [registrars] may be a bit reticent to kind of contact just to ask about that and may think, well, it's safe enough just to give the two doses, not gonna give much harm afterwards." *(Orthopaedic Consultant B)*
**Quotes illustrating the domains 'Social/professional role and identity' (5) and 'Social influences' (6)**		
**TDF domains**	**Key concepts**	**Illustrative quote**
Social/ professional role and identity	Roles and responsibilities for SAP decision making	"One of your questions was about whether we decide on what antibiotics. I don't consider that an anaesthetic decision. The choice about which antibiotic is up to surgeons." *(Anaesthetist F)*
		"I think it's the consultants who make the decisions. The input is given by the registrars because in the public system particularly, you don't know your patients. So, you get to be told by your registrars what the patient is like. And the registrars can give you suggestions saying 'That this person I think is not well' or whatever it is, 'I think we should give three doses' or whatever it is. And then the consultant makes the decision, of course, based on that." *(Orthopaedic Consultant H)*
Social influences	Relationship with seniors<br><br>Hierarchy	"I guess it's really the consultants that need to make the decision that they will change their practice and until they do, then the registrars won't be able to change their practice." *(Orthopaedic Registrar D)*
		"And the reality is unless the patient is actually at risk, I don't tend to bite the hand that feeds me, so to speak." *(Orthopaedic Registrar E)*

		"I've also had a similar conversation before saying 'Oh, this is what the actual eTG [electronic Therapeutic Guidelines] protocol or guidelines suggest'…it kind of just falls back to the 'Oh, this is what the consultant wants or this what the surgeon wants.'" *(Pharmacist D)*
**Quotes illustrating the domains 'Emotion' (7), 'Beliefs about consequences' (8) and 'Optimism' (9)**		
**TDF domains**	**Key concepts**	**Illustrative quote**
Emotion<br><br>Beliefs about consequences	Fear<br><br>Attitude towards postoperative doses	"We know the evidence suggests it doesn't make a difference, but sometimes we treat ourselves for the reasons we've talked about. There's a risk-benefit. Do we really want to prove that there is a difference by treating with a single dose and finding out later on that that patient had an infection when we could have given them three doses at very little risk to them and possibly at significant benefit to them? So, it's a difficult prospect." *(Orthopaedic Consultant D)*
		"You will always tend to say, 'Oh, it's better to give more than less and prevent infection'." *(Orthopaedic Consultant H)*
		"It could be that previously, they've had that one bad occasion and it's kind of set them up to always wanna [sic] do it all the time." *(Pharmacist D)*
	Attitude towards changing practice	"A lot of orthopaedics is dogma rather than evidence based so would there be a shift? I don't know in the near future, maybe in the distant future. I think you know that the thinking will be 'Why not just give the antibiotics, we've been doing this 24-hours' worth of antibiotics for years. There's very little side effects that you could get from giving the extra two doses of antibiotics'. So, if there's any form of protection, if you could even reduce the risk by 1% or you know however many percent, then why not?" *(Orthopaedic Registrar D)*
		"You question yourself as to why should you look at the guidelines when you [are] seemingly doing an ok job with the current set of protocols that you're anyway following? And unless there's some overwhelming evidence of whatever we're doing currently is totally wrong, I wouldn't want to look at those guidelines." *(Orthopaedic Consultant J)*

		"They're [HMO's and registrars] very often reluctant to cancel it because consultant's not at site and consultant orders are to just continue. So, it's a risk versus benefit I guess and also at the time of day, sometimes you just, one dose wouldn't hurt…kind of attitude." *(Pharmacist E)*
Optimism	Attitude towards changing practice	"If…you get an ideal circumstance, so as to say, then I suppose a single dose surgery would be reasonable." *(Orthopaedic Consultant J)*
		"I think if there's robust enough evidence to unequivocally support single dose of antibiotics over continued antibiotic use, then there could be a shift. And I definitely think there's precedent for that… I certainly think it's possible through education, research and antimicrobial stewardship that prescribing practices can improve." *(Orthopaedic Registrar E)*
**Quotes illustrating the domains 'Memory, attention and decision processes' (10), 'Behavioural regulation' (11) and 'Reinforcement' (12)**		
**TDF domains**	**Key concepts**	**Illustrative quotes**
Memory, attention and decision processes	Memory	"There have been times where they'll just leave it open ended without a particular cease date." *(Pharmacist D)*
	Factors that influence decision making<br><br>Patient factors<br><br>Collegial relationships	"Most upper limb fractures only use one dose. It's just this sort of horrible swollen ankle fractures, the diabetics, the ones that we know are more prone to infection, I'd be uncomfortable about. Even though I know, I'm aware the evidence suggests that one dose is fine." *(Orthopaedic Consultant G)*
		"I have colleagues who work in other hospitals. I have got registrars who rotate among different hospitals and of course there are our own AOA [Australian Orthopaedic Association] guidelines as well. So, these things definitely influence my practice." *(Orthopaedic Consultant H)*
Behavioural regulation	Audit and feedback	"I think audit is a very important part of everyone's practice. Self-audit as well as peer-reviewed audits are very important. And those are, I think, the most important driving factors that change our practice, the audit, because if we are doing something wrong that is harming the patient, we wanna [sic] know, we wanna [sic] change our practice so that it is better." *(Orthopaedic Consultant I)*

		"I think there are benefits in the process, definitely… And I think it would change, it would generate discussion and change practice for those that are not following the guidelines or evidence-based practice." *(Orthopaedic Registrar A)*
Reinforcement	Reminders	"It needs to be an ongoing thing, like we're not doing antibiotics until it becomes an Australia-wide or at least state-wide thing. It's gonna [sic] be a persistent problem every six months or every year when the registrars rotate." *(Orthopaedic Registrar D)*
		"We're prompted by our antimicrobial stewardship pharmacist to flag patients who are on postoperative antibiotics inappropriately and to box the next administration time for review. We are routinely reminded to do that. So, I feel like that's having a little bit of an impact." *(Pharmacist C)*
